# Supplementary material for: Polygenic risk score for type 2 diabetes shows context-dependent effects across populations
Source: Nat Commun. 2025 Oct 1;16:8632. doi: 10.1038/s41467-025-63546-4 (PMC12488948; doi:10.1038/s41467-025-63546-4)
Supplement: Supplementary file 1 — Supplementary Information [file 41467_2025_63546_MOESM1_ESM.pdf]

SUPPLEMENTARY INFORMATION

SUPPLEMENTARY FIGURES

Supplementary Figure 1. Density plots of the distribution of T2D PRS by self-reported race and ethnicity populations in the PAGE Study.

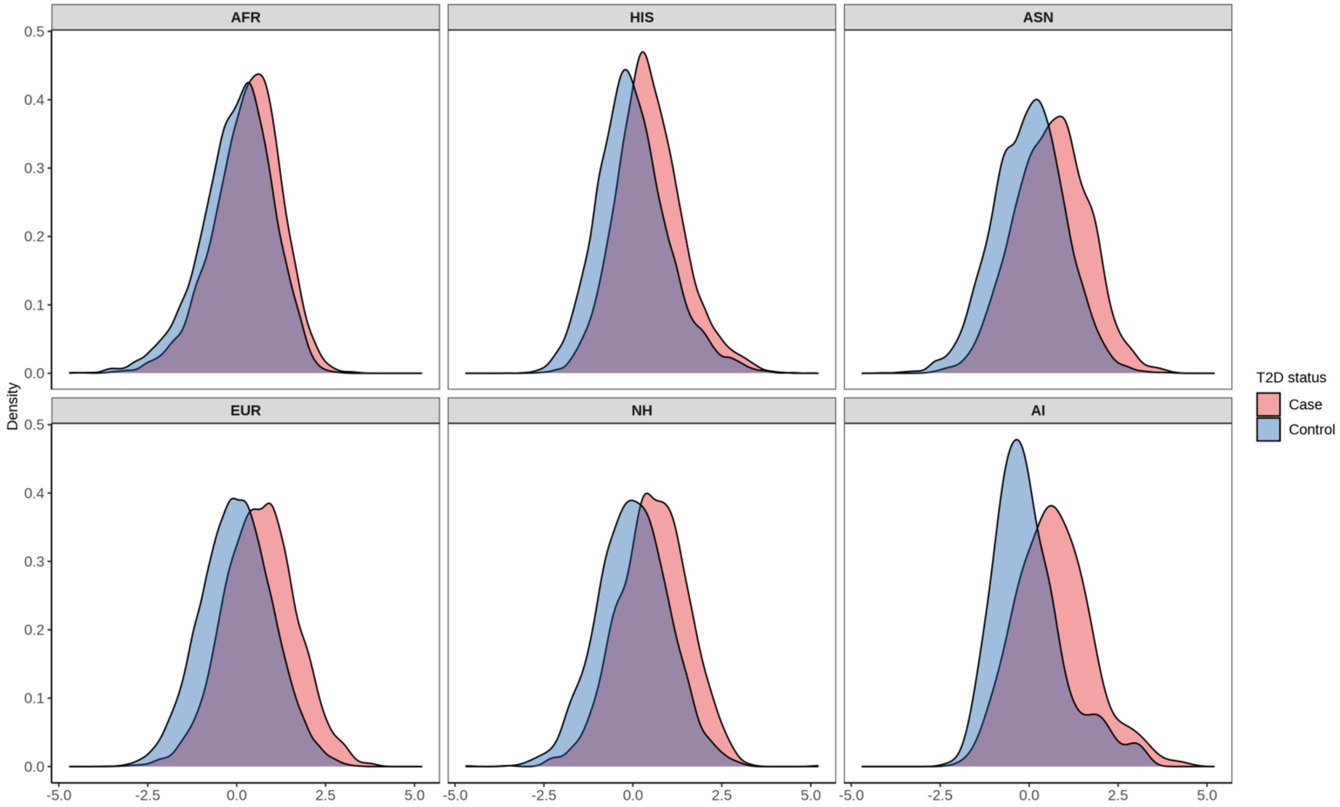

Supplementary Figure 2. **Association between each T2D PRS deciles and T2D risk by self-reported race and ethnicity populations in the PAGE Study, using the 40%-60% PRS category as the reference.** Data are presented as odds ratio (OR) with 95% confidence interval (CI) on a $\log_{10}$ scale.

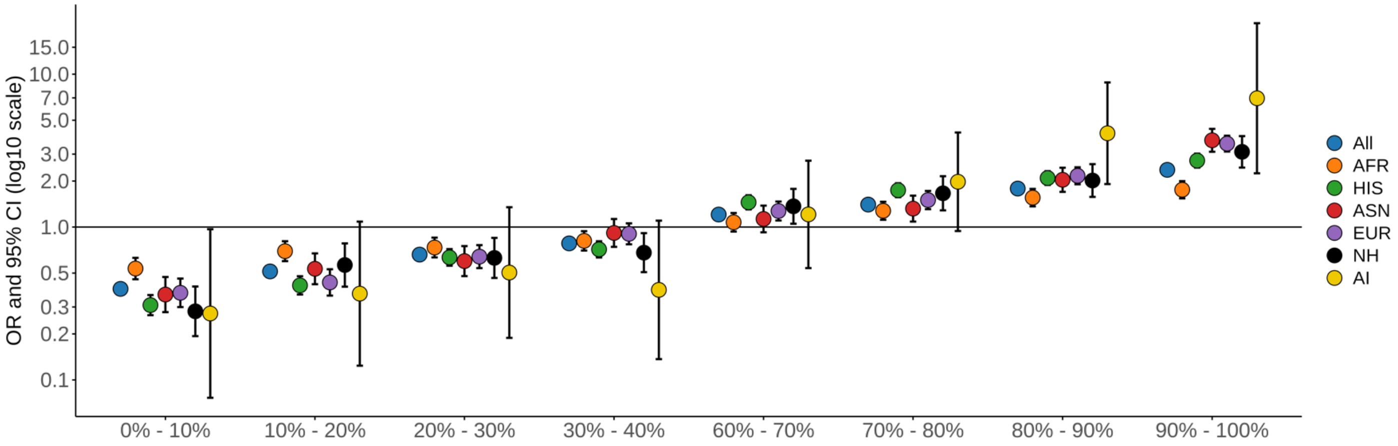

Supplementary Figure 3. Effect of the T2D PRS on T2D risk stratified by demographic, medical, and lifestyle and behavioral factors in the PAGE Study and meta-analyzed results from the additional biobanks and cohorts. P-values of heterogeneity from a two-sided Cochrane Q-test are indicated when differences were statistically significant (P < 0.05). **A** Demographic characteristics and medical history factors. **B** Behavioral and lifestyle factors. **C** Medication use. **D** Lipids. Data are presented as odds ratio (OR) with 95% confidence interval (CI). Sample sizes are provided in Supplementary Tables 10 and 11.

A

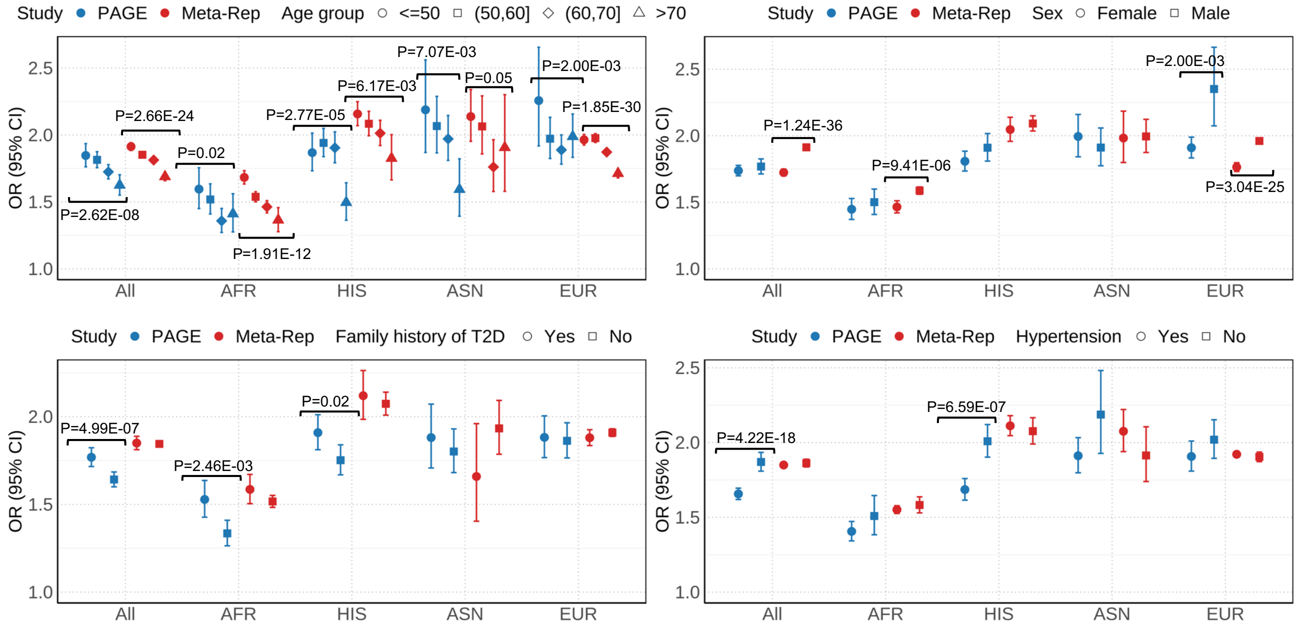

B

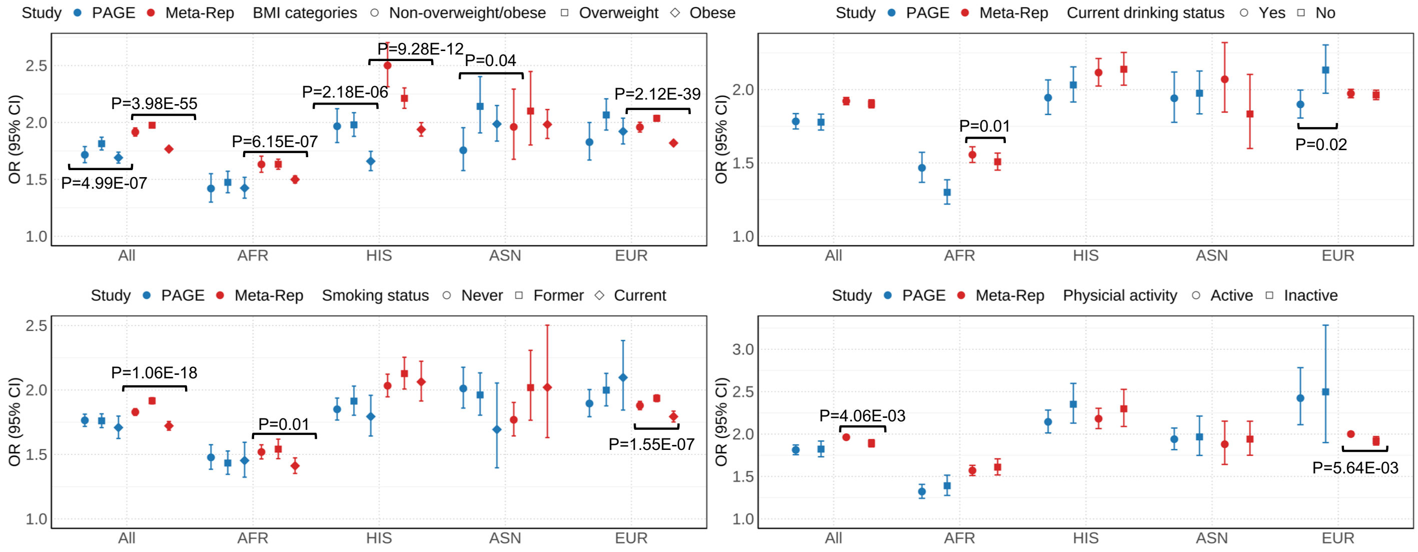

C

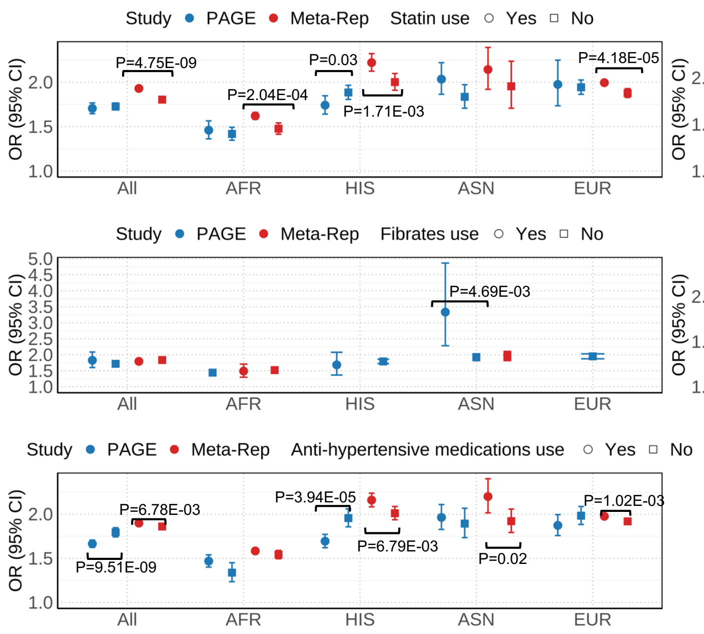
Study PAGE Meta-Rep Statin use Yes No
OR (95% CI)
P=4.75E-09
P=2.04E-04
P=0.03
P=1.71E-03
P=4.18E-05
All
AFR
HIS
ASN
EUR
Study PAGE Meta-Rep NSAID use Yes No
P=1.36E-04
P=0.02
P=0.03
P=6.43E-03
Study PAGE Meta-Rep Fibrates use Yes No
P=4.69E-03
Study PAGE Meta-Rep Lipid-lowering medicine use Yes No
P=3.10E-11
P=1.20E-03
P=0.03
P=0.01
P=4.69E-03
P=1.46E-05
Study PAGE Meta-Rep Anti-hypertensive medications use Yes No
P=6.78E-03
P=9.51E-09
P=3.94E-05
P=6.79E-03
P=0.02
P=1.02E-03

D

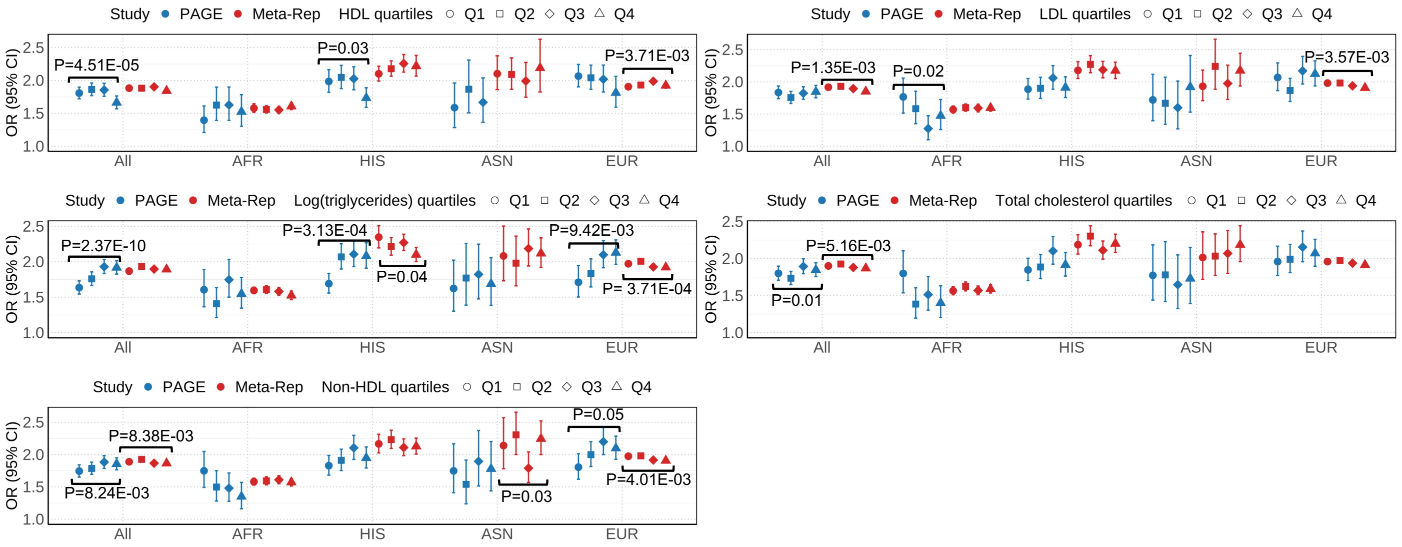
Study PAGE Meta-Rep HDL quartiles Q1 Q2 Q3 Q4
OR (95% CI)
P=4.51E-05
P=0.03
P=3.71E-03
All
AFR
HIS
ASN
EUR
Study PAGE Meta-Rep LDL quartiles Q1 Q2 Q3 Q4
P=1.35E-03
P=0.02
P=3.57E-03
Study PAGE Meta-Rep Log(triglycerides) quartiles Q1 Q2 Q3 Q4
P=2.37E-10
P=3.13E-04
P=0.04
P=9.42E-03
P= 3.71E-04
Study PAGE Meta-Rep Total cholesterol quartiles Q1 Q2 Q3 Q4
P=5.16E-03
P=0.01
Study PAGE Meta-Rep Non-HDL quartiles Q1 Q2 Q3 Q4
P=8.38E-03
P=8.24E-03
P=0.05
P=0.03
P=4.01E-03

Supplementary Figure 4. Effect of T2D PRS on diabetes-related traits meta-analyzed across PAGE and the additional biobanks and cohorts. The X-axis represents the beta estimates and 95% confidence intervals (CIs) of PRS for continuous traits and odds ratios and 95% CIs of PRS for binary traits. Solid circles indicate significant associations that passed the Bonferroni-adjusted P-value threshold of $P < 2.50 \times 10^{-3}$, while open circles indicate associations that were not significant. Panels show associations between the T2D PRS and **A** vascular diseases, **B** kidney function related traits, and **C** inflammatory biomarkers in T2D cases (left), controls (middle), and individuals with prediabetes (right). The units of continuous traits are listed in Supplementary Table 13. Sample sizes are provided in Supplementary Table 14. Results for glycemic and cardiometabolic traits are shown in Figure 5.

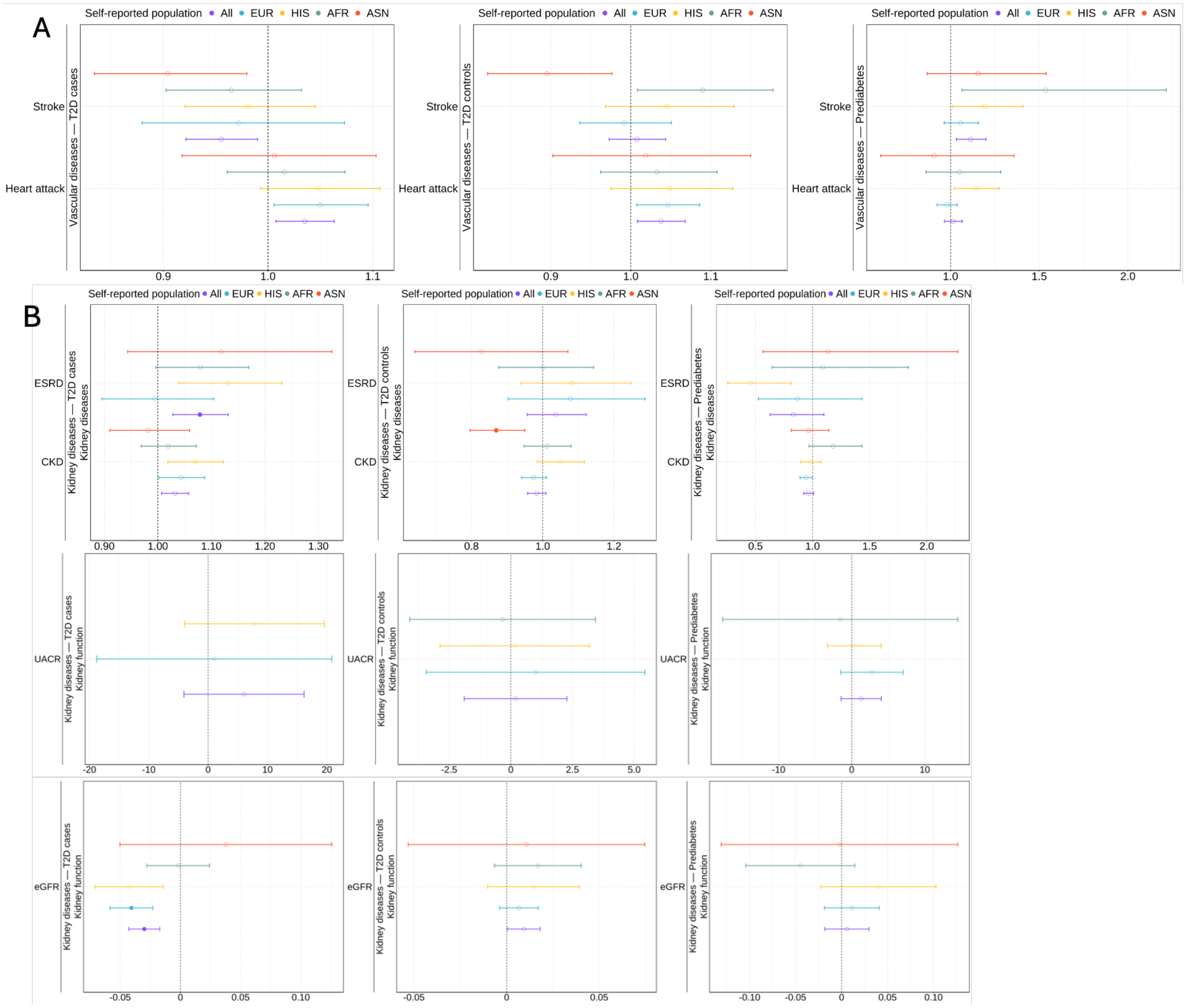

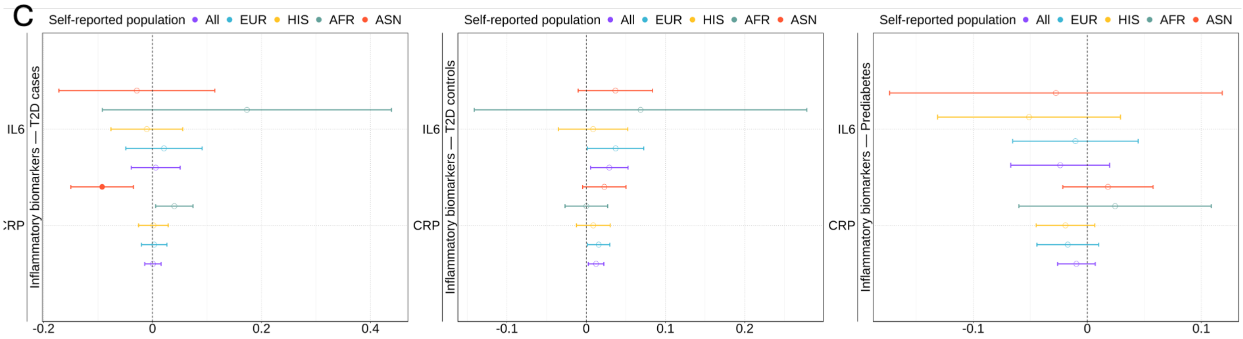
C
Self-reported population
All
EUR
HIS
AFR
ASN
Inflammatory biomarkers — T2D cases
Inflammatory biomarkers — T2D controls
Inflammatory biomarkers — Prediabetes
IL6
CRP
-0.2
0
0.2
0.4
-0.1
0.1
0.2

Supplementary Figure 5. T2D PRS PheWAS results meta-analyzed across all five biobanks by population. The X-axis represents phecodes color-coded by their corresponding phenotype category and is ordered from the category with the most to the category with the least significant hits. The Y-axis represents the $-\log_{10}$(p-value). The red horizontal line represents Bonferroni-adjusted p-value thresholds (EUR: $P < 2.76 \times 10^{-5}$, HIS: $P < 2.95 \times 10^{-5}$ AFR: $P < 2.81 \times 10^{-5}$and ASN: $P < 4.27 \times 10^{-5}$), and the blue horizontal line represents an unadjusted p-value threshold of $P < 0.05$. Upward triangles indicate positive associations, while downward triangles indicate negative associations. The top ten most significant associations from the endocrine/metabolic category are annotated, while the single most significant association from each other category is annotated. **A** EUR population. **B** HIS population **C** AFR population **D** ASN population.

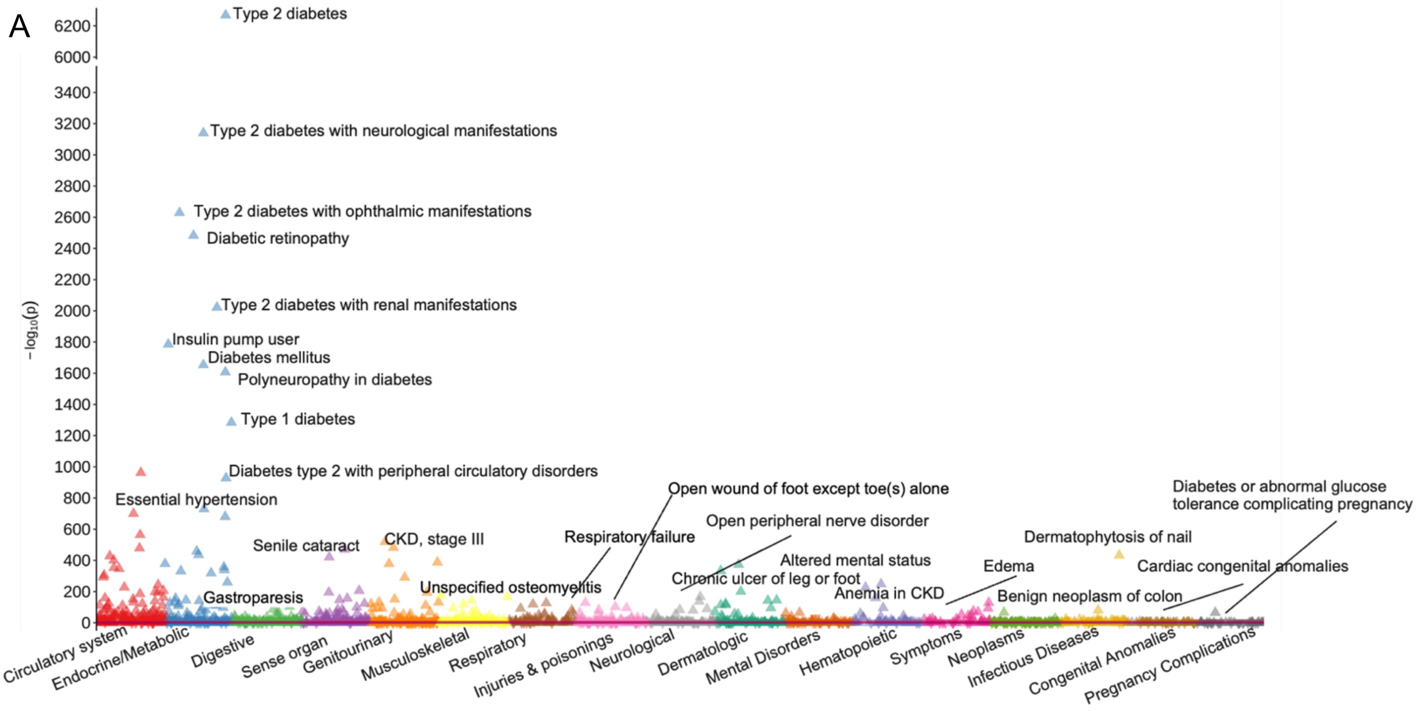

B

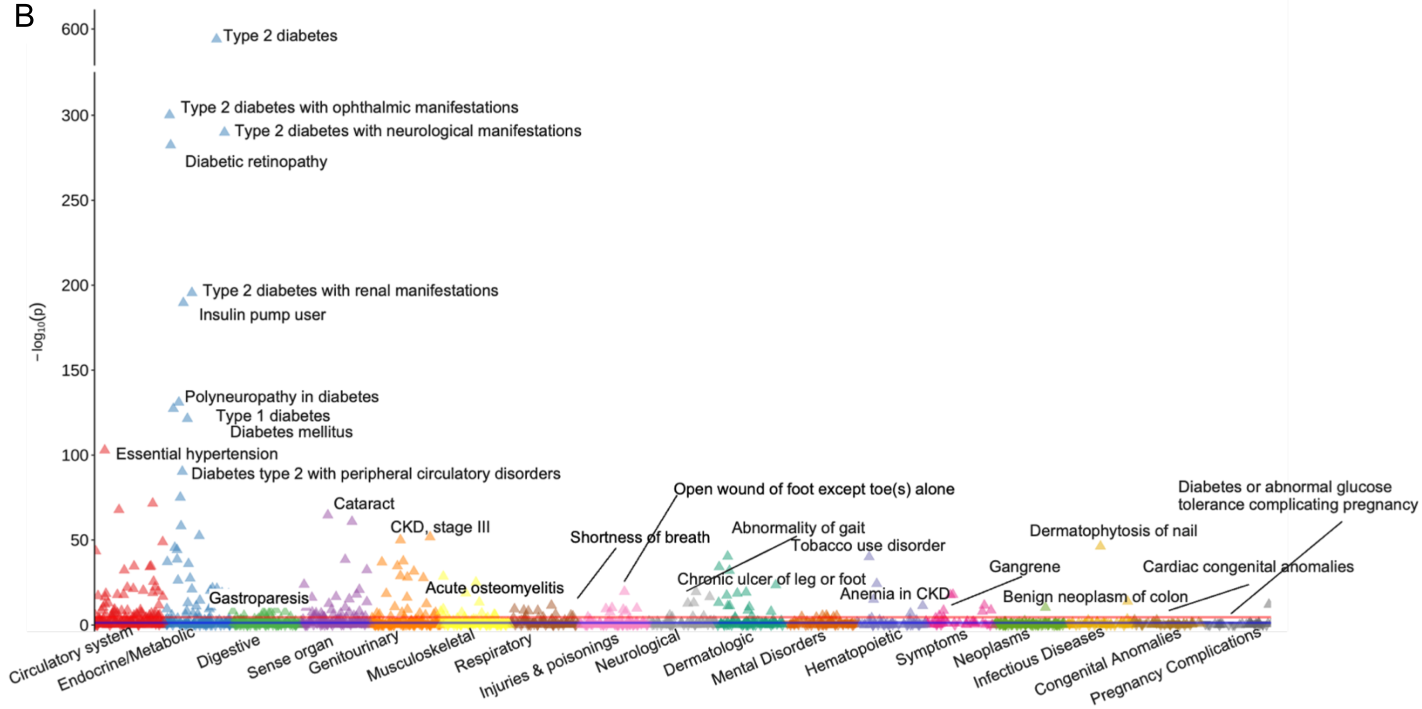

−log10(p)
Type 2 diabetes
Type 2 diabetes with ophthalmic manifestations
Type 2 diabetes with neurological manifestations
Diabetic retinopathy
Type 2 diabetes with renal manifestations
Insulin pump user
Polyneuropathy in diabetes
Type 1 diabetes
Diabetes mellitus
Essential hypertension
Diabetes type 2 with peripheral circulatory disorders
Cataract
CKD, stage III
Gastroparesis
Acute osteomyelitis
Open wound of foot except toe(s) alone
Shortness of breath
Abnormality of gait
Tobacco use disorder
Chronic ulcer of leg or foot
Anemia in CKD
Gangrene
Dermatophytosis of nail
Diabetes or abnormal glucose tolerance complicating pregnancy
Cardiac congenital anomalies
Benign neoplasm of colon
Circulatory system
Endocrine/Metabolic
Digestive
Sense organ
Genitourinary
Musculoskeletal
Respiratory
Injuries & poisonings
Neurological
Dermatologic
Mental Disorders
Hematopoietic
Symptoms
Neoplasms
Infectious Diseases
Congenital Anomalies
Pregnancy Complications

C

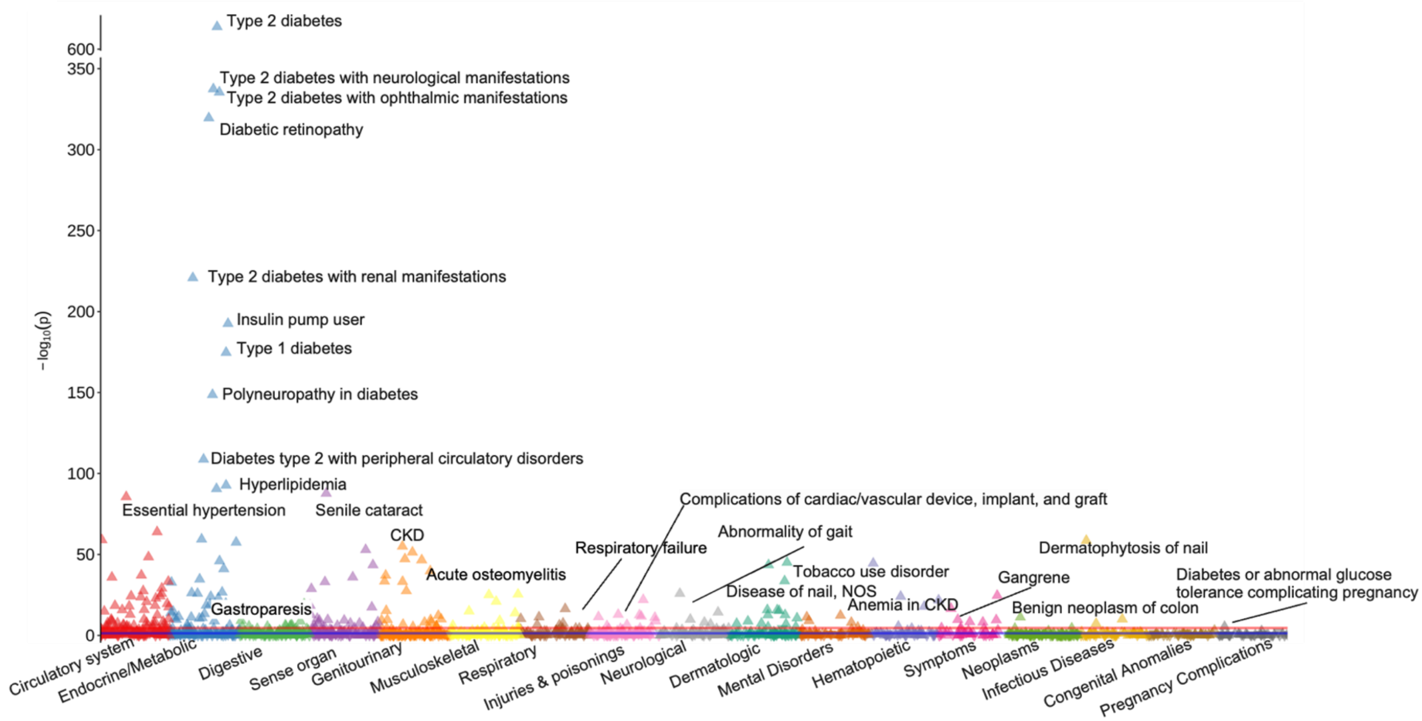

−log10(p)
Type 2 diabetes
Type 2 diabetes with neurological manifestations
Type 2 diabetes with ophthalmic manifestations
Diabetic retinopathy
Type 2 diabetes with renal manifestations
Insulin pump user
Type 1 diabetes
Polyneuropathy in diabetes
Diabetes type 2 with peripheral circulatory disorders
Hyperlipidemia
Essential hypertension
Senile cataract
CKD
Acute osteomyelitis
Gastroparesis
Complications of cardiac/vascular device, implant, and graft
Respiratory failure
Abnormality of gait
Tobacco use disorder
Disease of nail, NOS
Anemia in CKD
Gangrene
Dermatophytosis of nail
Benign neoplasm of colon
Diabetes or abnormal glucose tolerance complicating pregnancy
Circulatory system
Endocrine/Metabolic
Digestive
Sense organ
Genitourinary
Musculoskeletal
Respiratory
Injuries & poisonings
Neurological
Dermatologic
Mental Disorders
Hematopoietic
Symptoms
Neoplasms
Infectious Diseases
Congenital Anomalies
Pregnancy Complications

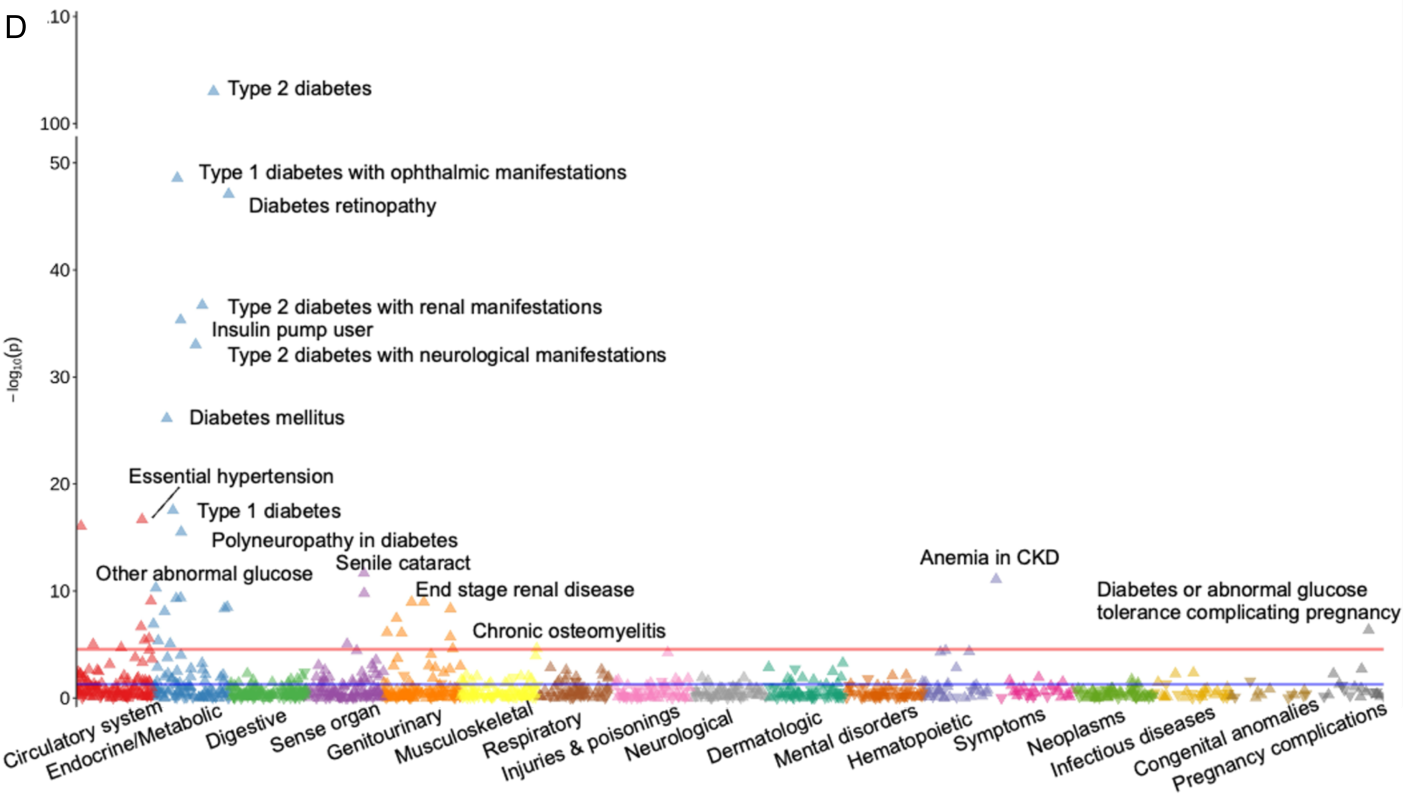
D
.10
100
50
40
30
20
10
0
−log10(p)
Type 2 diabetes
Type 1 diabetes with ophthalmic manifestations
Diabetes retinopathy
Type 2 diabetes with renal manifestations
Insulin pump user
Type 2 diabetes with neurological manifestations
Diabetes mellitus
Essential hypertension
Type 1 diabetes
Polyneuropathy in diabetes
Other abnormal glucose
Senile cataract
End stage renal disease
Chronic osteomyelitis
Anemia in CKD
Diabetes or abnormal glucose
tolerance complicating pregnancy
Circulatory system
Endocrine/Metabolic
Digestive
Sense organ
Genitourinary
Musculoskeletal
Respiratory
Injuries & poisonings
Neurological
Dermatologic
Mental disorders
Hematopoietic
Symptoms
Neoplasms
Infectious diseases
Congenital anomalies
Pregnancy complications

Description of the included studies, and definitions of T2D cases, individuals with prediabetes, and T2D controls

I. PAGE-participating studies

Atherosclerosis Risk in Communities (ARIC) Study[1] is a longitudinal cohort study designed to investigate the etiology of atherosclerosis and its clinical outcomes. Initiated in 1987, the ARIC study recruited participants between the ages of 45 and 65 from four U.S. communities: Forsyth County, NC; Jackson, MS; Washington County, MD; and Minneapolis, MN. Participants received an extensive examination of their medical, social and demographic profiles. The first four study visits occurred approximately every three years, followed by a gap of over 12 years before visit 5, with variable intervals between subsequent visits.

In the ARIC study, blood samples were collected during the baseline visit. Prevalent T2D was identified during the baseline examination if participants exhibited ≥8 h fasting blood glucose levels ≥126 mg/dL, or non-fasting glucose ≥200 mg/dL, self-reported a physician diagnosis of diabetes or "sugar in the blood," or reported current use of diabetes medication within the last two weeks. Participants were defined as T2D controls if they did not report using diabetes medication within the past two weeks and had fasting glucose levels <126 mg/dL.

The Icahn School of Medicine at Mount Sinai BioMe biobank in New York City (BioMe) is an electronic medical record (EMR)-linked biobank enriched for racially and ethnically diverse populations recruited from Mount Sinai Medical Center in upper Manhattan area, New York City since 2007. Data on anthropometrics, demographics, and medication use were derived from participants' EMR and a medical history questionnaire administered at baseline. (https://icahn.mssm.edu/research/ipm/programs/biome-biobank/facts).

BioMe implemented the eMERGE algorithm[2] to define T2D cases and controls. T2D cases were participants meeting any of the following criteria: 1) having International Classifiers of Disease (ICD-9-CM) codes of 250.x0 or 250.x2, except for 250.10 and 250.12, 2) on T2D medications and/or insulin, or 3) glucose > 200 mg/dl or HbA1c ≥ 6.5% laboratory results. Cases with a T2D diagnosis code were required to have either an abnormal laboratory test or a T2D medication prescription. Cases without a T2D diagnosis code were required to have documentation of both a prescription for a T2D medication and random glucose >200 mg/dl, or fasting glucose >125 mg/dl or HbA1c ≥6.5%. For patients treated with insulin alone, past prescription for a T2D medication or meeting specific criteria (no T1D diagnoses and ≥ 2 T2D diagnosis dates) were necessary.

T2D controls were defined by excluding those with any diabetes diagnosis (ICD-9-CM codes 250.xx), patients on insulin or any of diabetes medications, patients who used any diabetic supplies, and patients with any abnormal glucose (≥110 mg/dl) or HbA1c (≥6.0%) values. Patients with a family history of diabetes were also excluded. Controls were required to have ≥ 1 normal glucose measurement and ≥ 2 in-person clinician encounters.

Coronary Artery Risk Development in Young Adults (CARDIA)[3] is a longitudinal cohort study aims to identify factors that contribute to cardiovascular diseases in later life, and to better

understand the natural history of cardiovascular diseases over the adult life. 5,115 African American and European American participants aged 18 to 30 years were recruited from four urban communities: Birmingham, AL; Chicago, IL; Minneapolis, MN; and Oakland, CA in 1985-1986. In-person examinations occurred at 2, 5, 7, 10, 15, 20, 25, and 30 (2015-2016) years after baseline with annual telephone interviews to update health status and contact information.

T2D cases were identified at each examination using a combination of available information on ≥126 mg/dL fasting glucose, postchallenge (75 g) glucose ≥200 mg/dL, HbA1c ≥6.5%, or use of diabetes medications. Individuals diagnosed at <20 years old were excluded as they were considered to have T1D. T2D controls were defined as fasting glucose levels (<7.0 mmol/L and <126 mg/dL) at last available visit or no report of diabetes medication[4].

Hispanic Community Health Study/Study of Latinos (HCHS/SOL)[5,6] is a longitudinal cohort study of adults who were self-identified Hispanic/Latino and aged 18 to 74 years living in four study sites – Bronx, NY; Chicago, IL; Miami, FL; and San Diego, CA. The HCHS/SOL aims to determine the role of acculturation in the prevalence and incidence of diseases, and to identify influencing factors for the health of Hispanic/Latino populations. Participants received an extensive clinic exam and assessments to determine baseline risk factors during 2008-2022, had a second clinical visit during 2014-2017, and the third visit in January 2020 and concluded in January 2024.

T2D cases were defined as individuals with fasting time >8 h and fasting glucose levels ≥126 mg/dL, fasting ≤8 h and fasting glucose ≥200 mg/dL, post–oral glucose tolerance test glucose ≥200 mg/dL, HbA1c ≥6.5%, or on current treatment with antihyperglycemia medications. T2D controls were those with fasting time >8 h and fasting glucose levels <100 mg/dL, post–oral glucose tolerance test glucose <140 mg/dL, and HbA1c <5.6%[7].

Multiethnic Cohort Study (MEC)[8] is a longitudinal cohort study which follows over 215,000 residents of Hawai‘i and Los Angeles for development of cancer and other chronic diseases. It includes men and women of five main racial and ethnic groups, Japanese Americans, Native Hawaiians, African Americans, Latinos, and whites. The participants aged 45 to 75 years were recruited between 1993 and 1996. Biological specimens were collected from more than 70,000 MEC members from 2001 to 2005. Eight sub-studies from MEC were also included in this manuscript. They are MEC-AABC (African Ancestry Breast Cancer), MEC-AAPC (African Ancestry Prostate Cancer), MEC-JABC (Japanese Ancestry Breast Cancer), MEC-JAPC (Japanese Ancestry Prostate Cancer), MEC-LABC (Breast Cancer in Latinos), MEC-LAPC (Prostate Cancer in Latinos), MEC-SIGMA (the Slim Initiative in Genomic Medicine for the Americas), and MEC-HIBC (Native Hawaiian Breast Cancer).

T2D cases were defined as: 1) a self-report of diabetes on the baseline questionnaire, 2nd questionnaire or 3rd questionnaire, or 2) self-report of T2D medication at the time of blood draw, or 3) no diagnosis of T1D in the absence of a T2D diagnosis from the California Office of Statewide Health Planning and Development (OSHPD) for California Residents, or 4) individuals who were linked to the diabetes registries of Hawaii Medical Service Association (HMSA) or Kaiser Permanente (KPH) Hawaii health plans, or who were designated as diabetic

in the Chronic Conditions Data Warehouse of Medicare [9]. T2D controls were defined as: 1) no self-report of diabetes on any of the questionnaires while having completed a minimum of 2 of the 3 (79% of controls returned all 3 questionnaires); and 2) no use of medications for T2D at the time of blood draw; and 3) no T1D and T2D diagnosis from the OSHPD, HMSA or KPH registries. To preserve DNA for genetic studies of cancer in the MEC, subjects with an incident cancer diagnosis at time of selection for this study were excluded. Controls were frequency matched to cases on age at entry into the cohort (5-year age groups) and for Latinos, place of birth (U.S. vs. Mexico, South or Central America), oversampling African American, Native Hawaiian and European American controls to increase statistical power. For controls with glucose measured, individuals with >126 mg/dL were also excluded.

Women's Health Initiative (WHI)[10] is a longitudinal cohort study focuses on strategies for preventing heart disease, breast and colorectal cancer, and osteoporosis in postmenopausal women. The WHI study collected long-term data from 52,068 WHI women aged 50 – 79 years between 1993 and 1998 at 40 clinical centers across the U.S. Seven sub-studies from WHI were also included in this manuscript. They are WHI-GARNET(Genomics and Randomized Trials Network), WHI-HIPFX (the Hip Fracture GWAS), WHI-GECCO (the Genetics and Epidemiology of Colorectal Cancer Consortium), WHI-LLS (the Long Life Study), WHI-MOPMAP (the Modification of PM-Mediate Arrhythmogenesis in Population study), WHI-WHIMS (Women's Health Initiative Memory Study), and WHI-SHARe (SNP Health Association Resource).

T2D was documented at baseline by self-report in which each woman was asked whether she had ever been told that she had "sugar diabetes" by her physician. Incident T2D cases were identified during annual follow-up with self-administered questionnaires. A WHI diabetes confirmation study has demonstrated consistency between these medical inventories and incident and prevalent diabetes[11]. T2D controls were selected if participants reported no diabetes at baseline and did not self-report of medication to treat diabetes through their last follow-up.

II. Additional biobanks and cohorts

All of Us (AoU)[12] is a longitudinal cohort study aims to collect data from at least one million individuals across the United States, creating a diverse health database for epidemiological and genomic studies. Initiated in May 2018, AoU has recruited over 781,000 participants as of March 26, 2024.

We defined T2D cases in AoU using the following criteria: adults aged ≥ 25 years and 1) reported T2D diagnosis, or current T2D treatment and medication use, or 2) had T2D, disorder due to T2D, or had complications due to T2D at ≥2 occurrence dates; or 3) had fasting glucose ≥126 mg/dL, or random glucose ≥200 mg/dL, or HbA1c ≥6.5%, at ≥2 occurrence dates. We excluded 1) those did not have short read whole genome sequencing (WGS) data, 2) indicated T1D diagnosis in the survey, 3) had T1D or related complications and disorder at ≥2 occurrence dates, 4) had pregnancy related T1D/T2D at ≥2 occurrence dates. Individuals with prediabetes were defined as WGS participants who: 1) reported prediabetes diagnosis, treatment and medication use in the survey, or 2) had T1D at ≥2 occurrence dates, or 3) had 100-125 mg/dL fasting glucose, 5.7–6.4% HbA1c, or 140–199 mg/dL from glucose tolerance 2 hours panel at ≥2

occurrence dates. T2D controls were defined as WGS participants aged ≥40 years and did not meet the definitions of T2D cases and prediabetes.

BioVU Biobank (BioVU)[13] is a de-identified EHR-based biobank launched in 2007 at Vanderbilt University Medical Center (VUMC), a tertiary-care center at Nashville, TN. BioVU consents patients in the out-patient clinic environments at VUMC to have their leftover blood from clinical care be banked for future research. BioVU aims to provide a resource for studies of genotype-phenotype associations (https://victr.vumc.org/what-is-biovu/). Owing to the de-identification process all BioVU participants are deemed non-human subjects by the Institutional Review Board.

Three sets of criteria were used to define T2D cases. Case definition I: 1) any of the following ICD-9-CM codes—250.3, 250.32, 250.2, 250.22, 250.9, 250.92, 250.8, 250.82, 250.7, 250.72, 250.6, 250.62, 250.5, 250.52, 250.4, 250.42, 250, and 250.02—or equivalent ICD-10-CM codes and 2) use of non-insulin diabetes medication. Case definition II: 1) any of the above ICD-9 codes; 2) a glucose concentration greater than 200 mg/dL or a HbA1c level greater than 6.5%; and 3) no use of insulin medication. Case definition III: 1) use of non-insulin diabetes medication and 2) a glucose concentration greater than 200 mg/dL or a HbA1c level greater than 6.5%. The controls were defined as “record does not contain any of the following information”: 1) ICD-9/10-CM codes from the case definition or any of following ICD-9-CM codes—790.21, 790.22, 790.29, 791.5, 648.8, 277.7, and 250, or equivalent ICD-10-CM; 2) use of insulin medication; 3) use of non-insulin diabetes medication; 4) history of diabetes; and 5) a glucose concentration ≥110 mg/dL or a HbA1c level ≥6.0%[14].

Cameron County Hispanic Study (CCHC)[15] is a randomly-ascertained, community-based cohort of study that recruited more than 5,000 Mexican American participants in Brownsville (Cameron County), TX since 2004. CCHC participants were followed at five, 10, and 15 years. Demographic, lifestyle, clinical measurements, and biospecimens were collected at each visit. The analyses were restricted to individuals with no closer than 3rd degree genetic kinship with each other (N=3019) who had measured genotype.

T2D cases were defined as participants who had either 1) T2D diagnosis; or 2) T2D medication use; 3) or fasting blood glucose ≥126 mg/dL; or 4) HbA1C ≥ 6.5%. Individuals with prediabetes were defined as participants without a T2D diagnosis, no diabetes medication use, and either had a fasting blood glucose level ≥100 mg/dL and <126 mg/dL or a HbA1C level ≥ 5.7% and <6%. T2D controls were participants who had no T2D diagnosis, no diabetes medication use, had a fasting blood glucose level <100 mg/dL, and a HbA1C level < 5.7%.

Cebu Longitudinal Health and Nutrition Survey (CLHNS)[16] is an ongoing longitudinal study with a focus on infant feeding patterns among Filipino women who gave birth between May 1, 1983, and April 30, 1984 (https://cebu.cpc.unc.edu/). The Cebu study expanded its scope to investigate various health, demographic, and nutritional outcomes, including birth weight, infant feeding practices, maternal diet, and child spacing. The baseline survey included 3,327 pregnant women from the Metropolitan Cebu area, with subsequent surveys conducted bimonthly for 24 months. Follow-up surveys tracked the participants into adolescence and young adulthood,

aiming to understand the intergenerational effects on health and nutrition. Overnight fasting blood samples for biomarker measurement and DNA extraction were obtained in 2005.

T2D cases were defined as participants who were currently on T2D medication, and/or those with adjusted glucose level ≥7.0 mmol/L. Adjusted glucose was calculated by subtracting 0.97 mmol/l from the measured overnight fasting blood glucose level. Individuals with prediabetes were defined as participants who did not meet the definition of T2D cases and had a glucose adjusted level ≥100 mg/dL. T2D controls were participants who were not on T2D medication, and had overnight fasting blood glucose <7.0 mmol/L.

China Health and Nutrition Survey (CHNS)[17] is a nationwide longitudinal study aimed at investigating a range of economic, sociological, demographic, and health-related inquiries within the Chinese population. Utilizing a stratified probability sampling method with a multistage, random cluster design, counties and cities across nine diverse provinces (Guangxi, Guizhou, Heilongjiang, Henan, Hubei, Hunan, Jiangsu, Liaoning, and Shandong) were selected, stratified by income and urbanization according to State Statistical Bureau definitions. A total of 4,560 households from 228 communities were then randomly chosen from each stratum. CHNS began in 1989 with additional surveys completed in 1997, 2000, 2004, 2006, 2009, 2011, 2015, and 2018.

CHNS used the same definitions of T2D cases, individuals with prediabetes, and T2D controls as PAGE (refer to the Methods - T2D cases and controls definition section), except that CHNS does not have random glucose and OGTT measurements.

Colorado Center for Personalized Medicine Biobank (CCPM)[18] is a EHR linked biobank that was jointly developed by the University of Colorado Anschutz Medical Campus and University of Colorado Health (UCHealth) to serve as a unique, dual-purpose research and clinical resource accelerating personalized medicine. Began in September 2015, CCPM enrolled >200,000 participants who received medical care at UCHealth University of Colorado Hospital in Denver, CO.

T2D cases were determined through the use of phecode 250.2X sourced from the PheWAS catalog (https://phewascatalog.org/), which maps ICD-9 and ICD-10 codes from CCPM participants to specific phecodes. Case identification required the presence of at least two distinct separate encounters. Exclusions were then applied to identify T2D controls.

Greenlandic Study (Greenlandic) consists of two cross-sectional Greenlandic cohorts[19]: The Inuit Health in Transition (IHIT)[20] and the Greenland population study (B99)[21]. The B99 and the IHIT cohorts were collected as part of a general population health survey of the adult Greenlandic population during 1998–2001 and 2005–2010, respectively. Only participants living in Greenland were included. The Greenlandic population is of mainly Inuit genetic ancestry but also has recent gene flow from Europe (on average 25% European ancestry and 75% Inuit ancestry).

The Greenlandic participants underwent an OGTT in which blood samples were drawn after an overnight fast and after 2 h during a 75g OGTT. The Greenlandic studies used the same definitions of T2D cases, individuals with prediabetes, and T2D controls as PAGE (refer to the Methods - T2D cases and controls definition section).

Multi-Ethnic Study of Atherosclerosis Study (MESA)[22] is a prospective cohort study aimed at studying the prevalence, progression, determinants, and prognostic significance of subclinical cardiovascular disease in a sex-balanced, multiethnic, community-dwelling U.S. cohort. MESA was initiated in 1999 and recruited >6,000 participants aged 45-84 seen at clinics in Columbia University, New York; Johns Hopkins University, Baltimore; Northwestern University, Chicago; UCLA, Los Angeles; University of Minnesota, Twin Cities; and Wake Forest University, Winston Salem (https://www.mesa-nhlbi.org/)

MESA used the same definitions of T2D cases, individuals with prediabetes, and T2D controls as PAGE (refer to the Methods - T2D cases and controls definition section), except that MESA does not have HbA1c and OGTT measurements.

Michigan Genomics Initiative (MGI)[23] is a single health-system biobank comprising >91,000 participants with age ranging from 18 to over 90 years, recruited primarily during surgical encounters at Michigan Medicine. MGI recruitment began in 2012 with the goal of combining patient EHR data with corresponding genetic data to gain novel biomedical insights and to accelerate biomedical and precision health research at the University of Michigan.

T2D cases were identified using the PheWAS R package[24] to map ICD-10 codes to phecodes (250.2X), and excluded those had a T1D phecode (250.1X). T2D controls were all individuals other than T2D cases.

Million Veteran Program (MVP)[25–27] is a cohort of fully consented veterans from the United States military, gathered from more than 75 participating Department of Veterans Affairs (VA) medical facilities. Since recruitment efforts started in 2011, 1 million Veterans have joined MVP with genotype data on over 650,000 racially/ethnically diverse individuals. Each study participant contributed blood samples for DNA extraction and genotyping, in addition to completing surveys regarding their health status, lifestyle choices, and military service experiences. Informed consent is also obtained from all participants to access to their full EHR within the VA prior to and after enrollment including inpatient International Classification of Diseases (ICD9/10) diagnosis codes, Current Procedural Terminology (CPT) codes, clinical laboratory measurements, and reports of diagnostic imaging modalities. The EHR is continuously being integrated with MVP genomic data and access to these linked coded data is provided to approved investigators. The study received ethical and study protocol approval from the VA Central Institutional Review Board.

Prevalent T2D cases were identified using precedes 250.2X, requiring the presence of at least two separate encounters at or before enrollment. T2D controls were all individuals other than T2D cases.

Qatar Biobank (Qatar)[28] is a population-based research initiative that creates a repository of biological samples and information on health and lifestyle of Qatari citizens and long-term residents (https://www.qatarbiobank.org.qa/). The Qatar Biobank aims to collect extensive lifestyle, clinical, and biological information from up to 60,000 Qatari nationals and long-term residents (individuals living in the country for ≥15 years) aged ≥18 years (approximately one-fifth of all Qatari citizens). Consented participants underwent a comprehensive assessment at the Qatar Biobank facility in Doha, Qatar. The assessment consisted of a 5-stage interview and physical examination sequence, lasting approximately 3 hours on average. Participants provided biological samples (blood, urine, and saliva), which were analyzed at diagnostic laboratories.

T2D cases were identified if 1) they answered yes to the question "Has a doctor ever told you that you had or have diabetes?", or 2) if their HbA1c level was ≥6.5%, or 3) if fasting glucose level ≥126 mg/dL or non-fasting glucose level ≥200 mg/dL. Those took insulin were excluded as they were considered to have T1D. Additionally, individuals with prediabetes, defined as having fasting glucose levels between 100-125 mg/dL or HbA1c levels between 5.7 and 6.4, were removed, with precedence given to a diagnosis of diabetes over prediabetes. Furthermore, individuals diagnosed with T2D at age <32 years, suspected of having T1D, were also excluded. Any remaining individuals not meeting these criteria were designated as T2Dcontrols.

REasons for Geographic and Racial Differences in Stroke Study (REGARDS)[29] is a national observational study aimed at investigating factors contributing to stroke risk in adults aged 45 years or older. Between January 2003 and October 2007, 30,239 participants were recruited and underwent a telephone interview followed by an in-home physical examination. Assessments included traditional stroke risk factors such as blood pressure and cholesterol levels, as well as electrocardiograms of the heart. Participants are contacted every six months via phone to inquire about stroke symptoms, hospitalizations, and general health status.

T2D cases were defined at the baseline and second in-home visits as fasting glucose ≥126 mg/dL or a random glucose ≥200 mg/dL among those who did not fast, or self-reported use of insulin or oral diabetes medication. T2D controls were everyone else who were not missing relevant data to classify the phenotype.

Supplementary References

1. Wright JD, Folsom AR, Coresh J, et al. The ARIC (Atherosclerosis Risk In Communities) Study: JACC Focus Seminar 3/8. *J Am Coll Cardiol*. 2021;77(23):2939-2959. doi:10.1016/j.jacc.2021.04.035
2. Kho AN, Hayes MG, Rasmussen-Torvik L, et al. Use of diverse electronic medical record systems to identify genetic risk for type 2 diabetes within a genome-wide association study. *J Am Med Inform Assoc JAMIA*. 2012;19(2):212-218. doi:10.1136/amiajnl-2011-000439
3. Friedman GD, Cutter GR, Donahue RP, et al. Cardia: study design, recruitment, and some characteristics of the examined subjects. *J Clin Epidemiol*. 1988;41(11):1105-1116. doi:10.1016/0895-4356(88)90080-7
4. Bancks MP, Kershaw K, Carson AP, Gordon-Larsen P, Schreiner PJ, Carnethon MR. Association of Modifiable Risk Factors in Young Adulthood With Racial Disparity in Incident Type 2 Diabetes During Middle Adulthood. *JAMA*. 2017;318(24):2457-2465. doi:10.1001/jama.2017.19546
5. Sorlie PD, Avilés-Santa LM, Wassertheil-Smoller S, et al. Design and implementation of the Hispanic Community Health Study/Study of Latinos. *Ann Epidemiol*. 2010;20(8):629-641. doi:10.1016/j.annepidem.2010.03.015
6. LaVange LM, Kalsbeek W, Sorlie PD, et al. Sample Design and Cohort Selection in the Hispanic Community Health Study/Study of Latinos. *Ann Epidemiol*. 2010;20(8):642-649. doi:10.1016/j.annepidem.2010.05.006
7. Qi Q, Stilp AM, Sofer T, et al. Genetics of Type 2 Diabetes in U.S. Hispanic/Latino Individuals: Results From the Hispanic Community Health Study/Study of Latinos (HCHS/SOL). *Diabetes*. 2017;66(5):1419-1425. doi:10.2337/db16-1150
8. Kolonel LN, Henderson BE, Hankin JH, et al. A multiethnic cohort in Hawaii and Los Angeles: baseline characteristics. *Am J Epidemiol*. 2000;151(4):346-357. doi:10.1093/oxfordjournals.aje.a010213
9. Waters KM, Stram DO, Hassanein MT, et al. Consistent Association of Type 2 Diabetes Risk Variants Found in Europeans in Diverse Racial and Ethnic Groups. McCarthy MI, ed. *PLoS Genet*. 2010;6(8):e1001078. doi:10.1371/journal.pgen.1001078
10. Anderson G, Cummings S, Freedman LS, et al. Design of the Women's Health Initiative clinical trial and observational study. *Control Clin Trials*. 1998;19(1):61-109. doi:10.1016/S0197-2456(97)00078-0
11. Margolis KL, Lihong Qi null, Brzyski R, et al. Validity of diabetes self-reports in the Women's Health Initiative: comparison with medication inventories and fasting glucose measurements. *Clin Trials Lond Engl*. 2008;5(3):240-247. doi:10.1177/1740774508091749
12. All of Us Research Program Investigators, Denny JC, Rutter JL, et al. The "All of Us" Research Program. *N Engl J Med*. 2019;381(7):668-676. doi:10.1056/NEJMsr1809937
13. Roden D, Pulley J, Basford M, et al. Development of a Large-Scale De-Identified DNA Biobank to Enable Personalized Medicine. *Clin Pharmacol Ther*. 2008;84(3):362-369. doi:10.1038/clpt.2008.89
14. Ritchie MD, Denny JC, Crawford DC, et al. Robust Replication of Genotype-Phenotype Associations across Multiple Diseases in an Electronic Medical Record. *Am J Hum Genet*. 2010;86(4):560-572. doi:10.1016/j.ajhg.2010.03.003

15. Fisher-Hoch SP, Rentfro AR, Salinas JJ, et al. Socioeconomic status and prevalence of obesity and diabetes in a Mexican American community, Cameron County, Texas, 2004-2007. *Prev Chronic Dis*. 2010;7(3):A53.
16. Adair LS, Popkin BM, Akin JS, et al. Cohort Profile: The Cebu Longitudinal Health and Nutrition Survey. *Int J Epidemiol*. 2011;40(3):619-625. doi:10.1093/ije/dyq085
17. Popkin BM, Du S, Zhai F, Zhang B. Cohort Profile: The China Health and Nutrition Survey—monitoring and understanding socio-economic and health change in China, 1989–2011. *Int J Epidemiol*. 2010;39(6):1435-1440. doi:10.1093/ije/dyp322
18. Wiley LK, Shortt JA, Roberts ER, et al. Building a vertically integrated genomic learning health system: The biobank at the Colorado Center for Personalized Medicine. *Am J Hum Genet*. 2024;111(1):11-23. doi:10.1016/j.ajhg.2023.12.001
19. Moltke I, Fumagalli M, Korneliussen TS, et al. Uncovering the Genetic History of the Present-Day Greenlandic Population. *Am J Hum Genet*. 2015;96(1):54-69. doi:10.1016/j.ajhg.2014.11.012
20. Bjerregaard P. Inuit Health in Transition—Greenland survey 2005-2010. Population sample and survey methods. sif. Accessed March 27, 2024. https://www.sdu.dk/da/sif/rapporter/2011/inuit_health_in_transition
21. Bjerregaard P, Curtis T, Borch-Johnsen K, et al. Inuit health in Greenland: a population survey of life style and disease in Greenland and among Inuit living in Denmark. *Int J Circumpolar Health*. 2003;62(sup1):3-79. doi:10.3402/ijch.v62i0.18212
22. Bild DE, Bluemke DA, Burke GL, et al. Multi-Ethnic Study of Atherosclerosis: Objectives and Design. *Am J Epidemiol*. 2002;156(9):871-881. doi:10.1093/aje/kwf113
23. Zawistowski M, Fritsche LG, Pandit A, et al. The Michigan Genomics Initiative: A biobank linking genotypes and electronic clinical records in Michigan Medicine patients. *Cell Genomics*. 2023;3(2):100257. doi:10.1016/j.xgen.2023.100257
24. Carroll RJ, Bastarache L, Denny JC. R PheWAS: data analysis and plotting tools for phenome-wide association studies in the R environment. *Bioinformatics*. 2014;30(16):2375-2376. doi:10.1093/bioinformatics/btu197
25. Gaziano JM, Concato J, Brophy M, et al. Million Veteran Program: A mega-biobank to study genetic influences on health and disease. *J Clin Epidemiol*. 2016;70:214-223. doi:10.1016/j.jclinepi.2015.09.016
26. Hunter-Zinck H, Shi Y, Li M, et al. Genotyping Array Design and Data Quality Control in the Million Veteran Program. *Am J Hum Genet*. 2020;106(4):535-548. doi:10.1016/j.ajhg.2020.03.004
27. Taliun D, Harris DN, Kessler MD, et al. Sequencing of 53,831 diverse genomes from the NHLBI TOPMed Program. *Nature*. 2021;590(7845):290-299. doi:10.1038/s41586-021-03205-y
28. Al Kuwari H, Al Thani A, Al Marri A, et al. The Qatar Biobank: background and methods. *BMC Public Health*. 2015;15(1):1208. doi:10.1186/s12889-015-2522-7
29. Howard VJ, Cushman M, Pulley L, et al. The reasons for geographic and racial differences in stroke study: objectives and design. *Neuroepidemiology*. 2005;25(3):135-143. doi:10.1159/000086678

STUDY ACKNOWLEDGEMENTS

The **Population Architecture Using Genomics and Epidemiology (PAGE)** program is funded by the National Heart, Lung and Blood Institute (NHLBI), supported by R01HL151152.

The contents of this paper are solely the responsibility of the authors and do not necessarily represent the official views of the NIH. The PAGE consortium thanks the staff and participants of the PAGE studies for their important contributions. The listing of PAGE senior investigators can be found at http://www.pagestudy.org.

PAGE-participating studies

ARIC: The Atherosclerosis Risk in Communities Study has been funded in whole or in part with Federal funds from the National Heart, Lung, and Blood Institute, National Institutes of Health, Department of Health and Human Services, under Contract nos. (75N92022D00001, 75N92022D00002, 75N92022D00003, 75N92022D00004, 75N92022D00005). The authors thank the staff and participants of the ARIC study for their important contributions. The datasets used for the analyses described in this manuscript were obtained from dbGaP under accession phs000223. We also acknowledge R01 HL143885 for multiomics, metabolomics, obesity and cardiovascular disease research.

BioMe: The Mount Sinai BioMe Biobank is supported by The Andrea and Charles Bronfman Philanthropies and by Federal funds from the NIH (U01HG00638001; U01HG007417; X01HL134588). Furthermore, analyses were in part supported through the computational and data resources and staff expertise provided by Scientific Computing and Data at the Icahn School of Medicine at Mount Sinai and by the Clinical and Translational Science Awards (CTSA) grant UL1TR004419 from the National Center for Advancing Translational Sciences. Research reported in this publication was also supported by the Office of Research Infrastructure of the National Institutes of Health under award number S10OD026880 and S10OD030463. The content is solely the responsibility of the authors and does not necessarily represent the official views of the National Institutes of Health.

The data used for the analyses described in this manuscript were obtained from dbGaP under accession phs000925.

CARDIA: The Coronary Artery Risk Development in Young Adults Study (CARDIA) is conducted and supported by the National Heart, Lung, and Blood Institute (NHLBI) in collaboration with the University of Alabama at Birmingham (HHSN268201800005I & HHSN268201800007I), Northwestern University (HHSN268201800003I), University of Minnesota (HHSN268201800006I), and Kaiser Foundation Research Institute (HHSN268201800004I). CARDIA was also partially supported by the Intramural Research Program of the National Institute on Aging (NIA) and an intra-agency agreement between NIA and NHLBI (AG0005).. The data used for the analyses described in this manuscript were obtained from dbGaP under accession phs000236.We also acknowledge R01 HL143885 for multiomics, metabolomics, obesity and cardiovascular disease research.

HCHS/SOL: The Hispanic Community Health Study/Study of Latinos is a collaborative study supported by contracts from the National Heart, Lung, and Blood Institute (NHLBI) to the University of North Carolina (HHSN268201300001I / N01-HC-65233), University of Miami (HHSN268201300004I / N01-HC-65234), Albert Einstein College of Medicine (HHSN268201300002I / N01-HC-65235), University of Illinois at Chicago – HHSN268201300003I / N01-HC-65236 Northwestern Univ), and San Diego State University (HHSN268201300005I / N01-HC-65237). The following Institutes/Centers/Offices have contributed to the HCHS/SOL through a transfer of funds to the NHLBI: National Institute on Minority Health and Health Disparities, National Institute on Deafness and Other Communication Disorders, National Institute of Dental and Craniofacial Research, National Institute of Diabetes and Digestive and Kidney Diseases, National Institute of Neurological Disorders and Stroke, NIH Institution-Office of Dietary Supplements. The data used for the analyses described in this manuscript were obtained from dbGaP under accession phs000555.

MEC: The MEC study is funded through the National Cancer Institute (U01 CA164973). The datasets used for the analyses described in this manuscript were obtained from dbGaP under accession phs000220.

WHI: The WHI program is funded by the National Heart, Lung, and Blood Institute, National Institutes of Health, U.S. Department of Health and Human Services through contracts 75N92021D00001, 75N92021D00002, 75N92021D00003, 75N92021D00004, 75N92021D00005. The datasets used for the analyses described in this manuscript were obtained from dbGaP under accession phs000227.

Additional biobanks and cohorts

BioVU: Vanderbilt University Medical Center's BioVU projects are supported by numerous sources: institutional funding, private agencies, and federal grants. These include National Institute of Health funded Shared Instrumentation Grant S10OD017985, S10RR025141, and S10OD025092; Clinical and Translational Science Awards grants UL1TR002243, UL1TR000445, and UL1RR024975. Data curation and phenotyping in this project are supported by SFORN grant 17SFRN33520017 from the American Heart Association.

Ethics approval and consent to participate

BioVU Consent form is provided to patients in the outpatient clinic environments at VUMC. The consent states policies on data sharing and privacy and, upon consent, makes any blood leftover from clinical care eligible for BioVU banking. The VUMC Institutional Review Board oversees BioVU and approved this project. All data included in this study was de-identified and unlinked to any identifying information. This study was reviewed by the Vanderbilt University Medical Center IRB (IRB# 190418 and 170924) and designated as non-human subjects research. The research was conducted in accordance with the principles of the Declaration of Helsinki.

CCPM: List of consortium members:

- Heather D. Anderson, PhD – Department of Clinical Pharmacy, University of Colorado Skaggs School of Pharmacy and Pharmaceutical Sciences, Anschutz Medical Campus

- Christina L. Aquilante, PharmD – Department of Pharmaceutical Sciences, University of Colorado Skaggs School of Pharmacy and Pharmaceutical Sciences, Anschutz Medical Campus
- Kelsey Arbogast, BA, MB
- Christopher H. Arehart, BS
- Ian M. Brooks, PhD – Department of Biomedical Informatics, University of Colorado School of Medicine, Anschutz Medical Campus; Health Data Compass, Office of the Vice Chancellor for Health Affairs, Anschutz Medical Campus
- Tonya M. Brunetti, MS, PhD
- Judith Brutus-Lestin, MD, MPH – UCHealth Core Lab
- Elizabeth E. Burke – CARES Innovation Center, UCHealth, Anschutz Medical Campus
- Emily M. Casteel, MPA
- Joanne B. Cole, PhD – Department of Biomedical Informatics, University of Colorado School of Medicine, Anschutz Medical Campus
- Curtis R. Coughlin II, PhD – Department of Pediatrics, University of Colorado School of Medicine, Anschutz Medical Campus
- Kristy Crooks, PhD – Department of Pathology, University of Colorado School of Medicine, Anschutz Medical Campus
- Jacob Crawford, BS
- Erin Culver, BS
- Michelle N. Edelmann, PhD – Health Data Compass, Office of the Vice Chancellor for Health Affairs, Anschutz Medical Campus
- Matthew J. Fisher, MS
- Alan W. Franklin, MS
- Teresa C. Frye, MT ASCP, MBA
- Hunter George, BA
- Chris R. Gignoux, PhD, MS – Department of Biomedical Informatics, University of Colorado School of Medicine, Anschutz Medical Campus
- Elizabeth K. Gilliland, MS, MB
- Casey S. Greene, PhD – Department of Biomedical Informatics, University of Colorado School of Medicine, Anschutz Medical Campus
- Brooke Hawkes, MS
- Emily Hearst, MHSA – CARES Innovation Center, UCHealth, Anschutz Medical Campus
- Audrey E. Hendricks, PhD – Department of Biomedical Informatics, University of Colorado School of Medicine, Anschutz Medical Campus; Department of Mathematical and Statistical Sciences, College of Arts and Sciences, University of Colorado Denver Campus
- Randi K. Johnson, PhD, MPH – Department of Biomedical Informatics, University of Colorado School of Medicine, Anschutz Medical Campus; Department of Epidemiology, Colorado School of Public Health, Anschutz Medical Campus
- Colleen G. Julian, PhD – Department of Biomedical Informatics, University of Colorado School of Medicine, Anschutz Medical Campus
- Dave Kao, MD – Division of Cardiology, Department of Medicine, University of Colorado School of Medicine, Anschutz Medical Campus; CARE Innovation Center, UCHealth, Anschutz Medical Campus

- Iain Konigsberg, PhD – Department of Biomedical Informatics, University of Colorado School of Medicine, Anschutz Medical Campus
- Lisa Ku, MS, CGC – Hereditary Cancer Clinic, UCHealth, Anschutz Medical Campus
- Elizabeth L. Kudron, MD, MPH – Department of Biomedical Informatics, University of Colorado School of Medicine, Anschutz Medical Campus; Department of Pediatrics, University of Colorado School of Medicine, Anschutz Medical Campus
- Rashawnda Lacy, MS – Health Data Compass, Office of the Vice Chancellor for Health Affairs, Anschutz Medical Campus
- Ethan M. Lange, PhD – Department of Biomedical Informatics, University of Colorado School of Medicine, Anschutz Medical Campus
- Yee Ming Lee, PharmD – Department of Clinical Pharmacy, University of Colorado Skaggs School of Pharmacy and Pharmaceutical Sciences, Anschutz Medical Campus
- Joe A. Lesny, BA
- Meng Lin, PhD – Department of Biomedical Informatics, University of Colorado School of Medicine, Anschutz Medical Campus
- Jan T. Lowery, PhD, MPH
- Luciana B. Vargas, BS, MS – Department of Biomedical Informatics, University of Colorado School of Medicine, Anschutz Medical Campus
- Betzaida L. Maldonado, MS, PhD candidate – Department of Biomedical Informatics, University of Colorado School of Medicine, Anschutz Medical Campus
- Darcy Marceau, BA, MB, CM
- James L. Martin, PharmD, MPH
- Brianna L. Gates, MS
- David Mayer, BS – Department of Biomedical Informatics, University of Colorado School of Medicine, Anschutz Medical Campus
- Nicole L. McDaniel, PharmD – Department of Clinical Pharmacy, University of Colorado Skaggs School of Pharmacy and Pharmaceutical Sciences, Anschutz Medical Campus
- Andrew Monte, MD, PhD – University of Colorado School of Medicine; University of Colorado School of Pharmacy & Pharmaceutical Sciences; Rocky Mountain Poison & Drug Safety, Denver Health
- Ethan Moore, BA
- Ann Nadrash, PharmD, BCPS – Ambulatory Pharmacy Health Outcomes Pharmacy Department, UCHealth, Anschutz Medical Campus
- Jack Pattee, PhD – Department of Biostatistics and Informatics, Center for Innovative Design and Analysis, Anschutz Medical Campus
- Nikita Pozdeyev, MD, PhD – Department of Biomedical Informatics, University of Colorado School of Medicine, Anschutz Medical Campus; Division of Endocrinology, University of Colorado School of Medicine, Anschutz Medical Campus
- Alaa Radwan, MS – Department of Clinical Pharmacy, University of Colorado Skaggs School of Pharmacy and Pharmaceutical Sciences, Anschutz Medical Campus
- Nick Rafaels, MS
- Sridharan Raghavan, MD, PhD
- Neda Rasouli, MD – Division of Endocrinology, Department of Medicine, University of Colorado School of Medicine, Anschutz Medical Campus
- Elise L. Shalowitz, MS

- Hoda Sherif, BS
- Johnathan A. Shortt, PhD – Department of Biomedical Informatics, University of Colorado School of Medicine, Anschutz Medical Campus
- Adrian M. Stewart, MB, CM
- Kristen J. Sutton, PhD – Department of Biomedical Informatics, University of Colorado School of Medicine, Anschutz Medical Campus
- Carolyn T. Swartz, BSN, RN – UCHealth Epic IT Department
- Anna Tanaka, BA – CARES Innovation Center, UCHealth, Anschutz Medical Campus
- Matthew R.G. Taylor, MD, PhD
- Candace Teague, MT
- Emily B. Todd, MS, CGC – Department of Biomedical Informatics, University of Colorado School of Medicine, Anschutz Medical Campus
- Katy E. Trinkley, PharmD, PhD – Department of Family Medicine, University of Colorado School of Medicine, Anschutz Medical Campus
- Laura K. Wiley, PhD – Department of Biomedical Informatics, University of Colorado School of Medicine, Anschutz Medical Campus

CLHNS: We thank the entire staff of the Office of Population Studies (OPS) Foundation in Cebu for their long-term work on the CLHNS. The CLHNS was supported by US National Institutes of Health grants R01DK078150, TW005596, HL085144; pilot funds from RR020649, ES010126, and DK056350; and the Office of Population Studies Foundation in Cebu. Additional support for data analysis was provided by US NIH R01DK072193.

CHNS: Support for CHNS is provided by NIH, R01HD30880 and R01AG065357. With additional funding from R01HD38700, R01 DK104371 and P30 DK056350, R01 HL108427, D43TW009077, 2CHD050924 and P30 AG066615, Additional support from China-Japan Friendship Hospital, Ministry of Health for support for CHNS 2009, Chinese National Human Genome Center at Shanghai since 2009, and Beijing Municipal Center for Disease Prevention and Control since 2011. We thank the National Institute for Nutrition and Health, China Center for Disease Control and Prevention, Beijing Municipal Center for Disease Control and Prevention, and the Chinese National Human Genome Center at Shanghai.

Greenlandic Study: We acknowledge all the participants and staff of the Greenlandic health surveys. The studies were approved by the Scientific Ethics Committee in Greenland (project 505-42, 505-95, project 2011–13 (ref. no. 2011–056978), project 2017-5582, project 2015–22 (ref. no. 2015-16426), and project 2021-09) and was conducted in accordance with the Declaration of Helsinki, second revision. All participants gave written informed consent.

MESA: The MESA projects are conducted and supported by the National Heart, Lung, and Blood Institute (NHLBI) in collaboration with MESA investigators. Support for the Multi-Ethnic Study of Atherosclerosis (MESA) projects are conducted and supported by the National Heart, Lung, and Blood Institute (NHLBI) in collaboration with MESA investigators. Support for MESA is provided by contracts 75N92020D00001, HHSN268201500003I, N01-HC-95159, 75N92020D00005, N01-HC-95160, 75N92020D00002, N01-HC-95161, 75N92020D00003, N01-HC-95162, 75N92020D00006, N01-HC-95163, 75N92020D00004, N01-HC-95164, 75N92020D00007, N01-HC-95165, N01-HC-95166, N01-HC-95167, N01-HC-95168, N01-HC-

95169, UL1-TR-000040, UL1-TR-001079, UL1-TR-001420, UL1TR001881, DK063491, and R01HL105756. The authors thank the other investigators, the staff, and the participants of the MESA study for their valuable contributions. A full list of participating MESA investigators and institutes can be found at http://www.mesa-nhlbi.org. This study was also supported in part by the NHLBI contracts R01HL151855, R01DK081572 and U01HG011723.

MGI: The authors acknowledge the Michigan Genomics Initiative participants, Precision Health at the University of Michigan, the University of Michigan Medical School Central Biorepository, and the University of Michigan Advanced Genomics Core for providing data and specimen storage, management, processing, and distribution services, and the Center for Statistical Genetics in the Department of Biostatistics at the School of Public Health for genotype data curation, imputation, and management in support of the research reported in this publication.

VA Million Veteran Program (MVP): This research is based in part on data from the Million Veteran Program, Office of Research and Development, Veterans Health Administration, and was supported by award 5I01BX003362 (PIs: Chang, Tsao). This publication does not represent the views of the Department of Veterans Affairs or the United States Government.

Core Acknowledgements (May 2024)
MVP Program Office
- Sumitra Muralidhar, Ph.D., Program Director
  US Department of Veterans Affairs, 810 Vermont Avenue NW, Washington, DC 20420
- Jennifer Moser, Ph.D., Associate Director, Scientific Programs
  US Department of Veterans Affairs, 810 Vermont Avenue NW, Washington, DC 20420
- Jennifer E. Deen, B.S., Associate Director, Cohort & Public Relations
  US Department of Veterans Affairs, 810 Vermont Avenue NW, Washington, DC 20420

MVP Executive Committee
- Co-Chair: Philip S. Tsao, Ph.D.
  VA Palo Alto Health Care System, 3801 Miranda Avenue, Palo Alto, CA 94304
- Co-Chair: Sumitra Muralidhar, Ph.D.
  US Department of Veterans Affairs, 810 Vermont Avenue NW, Washington, DC 20420
- J. Michael Gaziano, M.D., M.P.H.
  VA Boston Healthcare System, 150 S. Huntington Avenue, Boston, MA 02130
- Elizabeth Hauser, Ph.D.
  Durham VA Medical Center, 508 Fulton Street, Durham, NC 27705
- Amy Kilbourne, Ph.D., M.P.H.
  VA HSR&D, 2215 Fuller Road, Ann Arbor, MI 48105
- Michael Matheny, M.D., M.S., M.P.H.
  VA Tennessee Valley Healthcare System, 1310 24th Ave. South, Nashville, TN 37212
- Dave Oslin, M.D.
  Philadelphia VA Medical Center, 3900 Woodland Avenue, Philadelphia, PA 19104
- Deepak Voora, MD
  Durham VA Medical Center, 508 Fulton Street, Durham, NC 27705

MVP Co-Principal Investigators

- J. Michael Gaziano, M.D., M.P.H.
  VA Boston Healthcare System, 150 S. Huntington Avenue, Boston, MA 02130
- Philip S. Tsao, Ph.D.
  VA Palo Alto Health Care System, 3801 Miranda Avenue, Palo Alto, CA 94304

MVP Core Operations

- Jessica V. Brewer, M.P.H., Director, MVP Cohort Operations
  VA Boston Healthcare System, 150 S. Huntington Avenue, Boston, MA 02130
- Mary T. Brophy M.D., M.P.H., Director, VA Central Biorepository
  VA Boston Healthcare System, 150 S. Huntington Avenue, Boston, MA 02130
- Kelly Cho, M.P.H, Ph.D., Director, MVP Phenomics
  VA Boston Healthcare System, 150 S. Huntington Avenue, Boston, MA 02130
- Lori Churby, B.S., Director, MVP Regulatory Affairs
  VA Palo Alto Health Care System, 3801 Miranda Avenue, Palo Alto, CA 94304
- Scott L. DuVall, Ph.D., Director, VA Informatics and Computing Infrastructure (VINCI)
  VA Salt Lake City Health Care System, 500 Foothill Drive, Salt Lake City, UT 84148
- Saiju Pyarajan Ph.D., Director, Data and Computational Sciences
  VA Boston Healthcare System, 150 S. Huntington Avenue, Boston, MA 02130
- Robert Ringer, Pharm.D., Director, VA Albuquerque Central Biorepository
  New Mexico VA Health Care System, 1501 San Pedro Drive SE, Albuquerque, NM 87108
- Luis E. Selva, Ph.D., Director, MVP Biorepository Coordination
  VA Boston Healthcare System, 150 S. Huntington Avenue, Boston, MA 02130
- Shahpoor (Alex) Shayan, M.S., Director, MVP PRE Informatics
  VA Boston Healthcare System, 150 S. Huntington Avenue, Boston, MA 02130
- Brady Stephens, M.S., Principal Investigator, MVP Information Center
  Canandaigua VA Medical Center, 400 Fort Hill Avenue, Canandaigua, NY 14424
- Stacey B. Whitbourne, Ph.D., Director, MVP Cohort Development and Management
  VA Boston Healthcare System, 150 S. Huntington Avenue, Boston, MA 02130

MVP Publications and Presentations Committee

- Co-Chair: Themistocles L. Assimes, M.D., Ph. D
  VA Palo Alto Health Care System, 3801 Miranda Avenue, Palo Alto, CA 94304
- Co-Chair: Adriana Hung, M.D.; M.P.H
  VA Tennessee Valley Healthcare System, 1310 24th Ave. South, Nashville, TN 37212
- Co-Chair: Henry Kranzler, M.D.
  Philadelphia VA Medical Center, 3900 Woodland Avenue, Philadelphia, PA 19104

Qatar: The authors would like to acknowledge the Qatar BioBank (QBB) and the Qatar Genome Project (QGP).

REGARDS: This research project is supported by cooperative agreement U01 NS041588 co-funded by the National Institute of Neurological Disorders and Stroke (NINDS) and the National Institute on Aging (NIA), National Institutes of Health, Department of Health and Human Service. The content is solely the responsibility of the authors and does not necessarily represent

the official views of the NINDS or the NIA. Representatives of the NINDS were involved in the review of the manuscript but were not directly involved in the collection, management, analysis or interpretation of the data. The authors thank the other investigators, the staff, and the participants of the REGARDS study for their valuable contributions. A full list of participating REGARDS investigators and institutions can be found at: https://www.uab.edu/soph/regardsstudy/.
