# Supplementary material for: Polygenic risk score for type 2 diabetes shows context-dependent effects across populations
Source: Nat Commun. 2025 Oct 1;16:8632. doi: 10.1038/s41467-025-63546-4 (PMC12488948; doi:10.1038/s41467-025-63546-4)
Supplement: Supplementary file 2 — Description of Additional Supplementary Files [file 41467_2025_63546_MOESM2_ESM.pdf]

Description of Additional Supplementary Files

Supplementary Data 1. Sample sizes of the PAGE Study and additional biobanks and cohorts by population, study, T2D status, and genotyping array platform.

Supplementary Data 2. Detailed definitions of population descriptors in the PAGE Study and additional biobanks and cohorts.

Supplementary Data 3. Information on the four PRS methods evaluated in this study.

Supplementary Data 4. Performance of T2D PRS across self-identified populations: Area under the curve (AUC) for four T2D PRS methods in PAGE.

Supplementary Data 5. Performance of T2D PRS across self-identified populations: Association between each SD unit increase in the Ge et al. T2D PRS with T2D risk in PAGE and the additional biobanks and cohorts.

Supplementary Data 6. Association between PRS and mean age at T2D diagnosis in All of Us.

Supplementary Data 7. Definitions and subgroup categories for stratification analyses.

Supplementary Data 8. Summary of the effect of the T2D PRS on T2D risk stratified by demographic, medical, and lifestyle and behavioral factors.

Supplementary Data 9. Effect of the T2D PRS on T2D risk stratified by demographic, medical, and lifestyle and behavioral factors meta-analyzed across PAGE and the additional biobanks and cohorts.

Supplementary Data 10. Effect of the T2D PRS on T2D risk stratified by demographic, medical, and lifestyle and behavioral factors separately in PAGE and the additional biobanks and cohorts.

Supplementary Data 11. Effect of the T2D PRS on T2D risk stratified by demographic, medical, and lifestyle and behavioral factors meta-analyzed across additional biobanks and cohorts (not including PAGE).

Supplementary Data 12. Interaction results between PRS and demographic, medical, and lifestyle and behavioral factors in PAGE.

Supplementary Data 13. Units of outcomes and covariates adjusted for association analyses.

Supplementary Data 14. Effect of T2D PRS on diabetes-related traits meta-analyzed across PAGE and the additional biobanks and cohorts.

Supplementary Data 15. Effect of T2D PRS on diabetes-related traits separately in PAGE and the additional biobanks and cohorts.

Supplementary Data 16. Effect of T2D PRS on diabetes-related traits meta-analyzed across additional biobanks and cohorts (not including PAGE).

Supplementary Data 17. T2D PRS PheWAS results meta-analyzed across all populations and by population.
