# Supplementary material for: Polygenic risk score for type 2 diabetes shows context-dependent effects across populations
Source: Nat Commun. 2025 Oct 1;16:8632. doi: 10.1038/s41467-025-63546-4 (PMC12488948; doi:10.1038/s41467-025-63546-4)
Supplement: Supplementary file 4 — Reporting Summary [file 41467_2025_63546_MOESM4_ESM.pdf]

nature portfolio

Corresponding author(s): Boya Guo and Burcu Darst

Last updated by author(s): Apr 15, 2025

Reporting Summary

Nature Portfolio wishes to improve the reproducibility of the work that we publish. This form provides structure for consistency and transparency in reporting. For further information on Nature Portfolio policies, see our Editorial Policies and the Editorial Policy Checklist.

Statistics

For all statistical analyses, confirm that the following items are present in the figure legend, table legend, main text, or Methods section.

n/a	Confirmed	
☐	☒	The exact sample size (*n*) for each experimental group/condition, given as a discrete number and unit of measurement
☐	☒	A statement on whether measurements were taken from distinct samples or whether the same sample was measured repeatedly
☐	☒	The statistical test(s) used AND whether they are one- or two-sided<br>*Only common tests should be described solely by name; describe more complex techniques in the Methods section.*
☐	☒	A description of all covariates tested
☐	☒	A description of any assumptions or corrections, such as tests of normality and adjustment for multiple comparisons
☐	☒	A full description of the statistical parameters including central tendency (e.g. means) or other basic estimates (e.g. regression coefficient) AND variation (e.g. standard deviation) or associated estimates of uncertainty (e.g. confidence intervals)
☐	☒	For null hypothesis testing, the test statistic (e.g. *F*, *t*, *r*) with confidence intervals, effect sizes, degrees of freedom and *P* value noted<br>*Give P values as exact values whenever suitable.*
☒	☐	For Bayesian analysis, information on the choice of priors and Markov chain Monte Carlo settings
☒	☐	For hierarchical and complex designs, identification of the appropriate level for tests and full reporting of outcomes
☐	☒	Estimates of effect sizes (e.g. Cohen's *d*, Pearson's *r*), indicating how they were calculated

Our web collection on statistics for biologists contains articles on many of the points above.

Software and code

Policy information about availability of computer code

Data collection	No software was used for data collection.
Data analysis	Imputation was done using the NHLBI Trans-Omics for Precision Medicine (TOPMed) Imputation Server (https://imputation.biodatacatalyst.nhlbi.nih.gov/#!) with TOPMed-r2 as reference panel, Eagle v2.4 (https://alkesgroup.broadinstitute.org/Eagle/) for phasing, and Minimac4 (https://genome.sph.umich.edu/wiki/Minimac4) for imputation.<br><br>Polygenic risk score modeling was performed using the PRS-CSX (https://github.com/getian107/PRScsx) and PLINK 2.0 (https://www.cog-genomics.org/plink/2.0/).<br><br>Other statistical analyses and data visualizations were conducted in R version 4.1.2 (https://www.r-project.org/) with R packages rtracklayer (https://bioconductor.org/packages/release/bioc/html/rtracklayer.html) for liftover between genome reference builds, meta (https://cran.r-project.org/web/packages/meta/index.html) and metafor (https://www.metafor-project.org/doku.php/metafor) for meta-analysis, PheWAS (https://github.com/PheWAS/PheWAS) for PheWAS analyses, and ggplot2 (https://ggplot2.tidyverse.org/) for data visualizations.

For manuscripts utilizing custom algorithms or software that are central to the research but not yet described in published literature, software must be made available to editors and reviewers. We strongly encourage code deposition in a community repository (e.g. GitHub). See the Nature Portfolio guidelines for submitting code & software for further information.

Data

Policy information about availability of data

All manuscripts must include a data availability statement. This statement should provide the following information, where applicable:

- Accession codes, unique identifiers, or web links for publicly available datasets
- A description of any restrictions on data availability
- For clinical datasets or third party data, please ensure that the statement adheres to our policy

The Ge et al. T2D PRS and the T2D PRS of 582 variants are available on the PGS Catalog (https://www.pgscatalog.org; PGS IDs: PGS002308 and PGS000804, respectively). The newly generated PRS in this study will be published on the PGS Catalog. GWAS summary statistics from the Mahajan et al. 2022 study are available on the DIAGRAM consortium website (http://diagram-consortium.org). GWAS summary statistics from the Vujkovic et al., 2020 study are available on dbGaP (accession numbers pha004945.1, pha004943.1, pha004946.1, and pha004944.1, respectively). LD matrices used for PRS-CSx are available at https://github.com/getian107/PRScsx.

Research involving human participants, their data, or biological material

Policy information about studies with human participants or human data. See also policy information about sex, gender (identity/presentation), and sexual orientation and race, ethnicity and racism.

Reporting on sex and gender

We used self-reported sex at birth in this study.

Reporting on race, ethnicity, or other socially relevant groupings

Self-identified race and ethnicity (SIRE) population descriptors were used across studies, with the goal of studying how PRS performance may vary across the contexts of SIRE. Although other sociodemographic and environmental factors are expected to be more informative than SIRE, this investigation is limited by the variables that are currently available across all included studies. It is important to note that SIRE does not serve as a proxy for genetic ancestry, holds no biological basis, and does not imply a biological explanation for health disparities.

We standardized population descriptors across all studies in the analysis by using consistent acronyms. Specifically, AFR refers to African, African American, or Black participants; AI refers to American Indian, Alaskan Native, or Native American; ASN includes Asian American, East or Southeast Asian (e.g., from China, Japan, Korea, Indonesia, the Philippines), as well as South Asian/Indian (e.g., from India, Pakistan); EUR refers to non-Hispanic White American or European individuals; Greenlandic refers to individuals of Greenlandic or mixed Greenlandic-Danish heritage; HIS represents Hispanic/Latino American participants; MENAQ encompasses Qatari and Arab American individuals; and NH refers to Native Hawaiian or Other Pacific Islander. The Greenlandic population was considered separate from others as this population represents substantial Inuit ancestry. Detailed definitions of population descriptors used in each study are provided in Table S15.

Population characteristics

An overview of the study design is provided in Figure 1. In total, 82,944 participants from PAGE and 835,241 participants from 13 additional biobanks and cohorts were included in this study: 244,637 prevalent T2D cases, 637,891 controls, and 35,657 individuals with prediabetes. This included individuals who self-reported as African, African American, or Black (AFR); American Indian, Alaskan Native, or Native American (AI); Asian American, East of Southeast Asian, or South Asian/Indian (ASN); White or European American (EUR); Greenlandic; Hispanic/Latino American (HIS); Middle Eastern, North African, or Qatari (MENAQ); and Native Hawaiian or Other Pacific Islander (NH) (Table 1, Table S1, Methods, and Supplemental Information for population descriptor details).

Recruitment

The PAGE Study was developed to conduct genetic epidemiological research in ancestrally diverse populations within the United States. PAGE includes individuals from the following ongoing population-based biobanks and cohorts: Atherosclerosis Risk in Communities (ARIC), the Icahn School of Medicine at Mount Sinai BioMe biobank in New York City (BioMe), Coronary Artery Risk Development in Young Adults Study (CARDIA), Hispanic Community Health Study/Study of Latinos (HCHS/SOL), Multiethnic Cohort Study (MEC), and Women’s Health Initiative (WHI). A detailed description of PAGE has been described, with additional detail summarized in the Supplemental Information and Table S1.

Analyses were also conducted in 13 additional biobanks and cohorts that were not included in the discovery GWAS summary statistics of the best performing T2D PRS. Studies included All of Us (AoU), BioMe (participants were non-overlapping with those from BioMe included in PAGE), BioVU Biobank (BioVU), Cameron County Hispanic Study (CCHC), Cebu Longitudinal Health and Nutrition Survey (CLHNS), China Health and Nutrition Survey (CHNS), Colorado Center for Personalized Medicine Biobank (CCPM), Greenlandic Health Surveys (Greenlandic), Multi-Ethnic Study of Atherosclerosis Study (MESA)63, Michigan Genomics Initiative (MGI), Million Veteran Program (MVP), Qatar Biobank (Qatar), and REasons for Geographic and Racial Differences in Stroke Study (REGARDS) (Table S1).

Ethics oversight

The institutional review board at each of the study sites approved the study protocols, and written informed consent was obtained from all participants.

Note that full information on the approval of the study protocol must also be provided in the manuscript.

Field-specific reporting

[illegible]

Life sciences study design

All studies must disclose on these points even when the disclosure is negative.

Sample size	In total, 82,944 participants from PAGE and 835,241 participants from 13 additional biobanks and cohorts were included in this study: 244,637 prevalent T2D cases, 637,891 controls, and 35,657 individuals with prediabetes.
Data exclusions	Participants were excluded if they did not meet the definitions of either T2D cases, T2D controls or prediabetes. In PAGE, participants were classified as T2D cases if they met at least one of the following criteria: adults ≥18 years old with 1) a T2D diagnosis by a physician/medical professional or use of medication for treatment of diabetes, 2) a fasting (≥8 hour) plasma glucose ≥126 mg/dl, 3) a random glucose ≥200 mg/dl, or 4) an HbA1c ≥48 mmol/mol (6.5%). Cases were restricted to those with either an age at diagnosis or most recent age at glucose or HbA1c draw ≥25 years to avoid misclassifying T1D cases as T2D cases. Individuals with prediabetes were defined as adults ≥18 years old who did not meet the definition of T2D and with 1) a fasting plasma glucose between 100-125 mg/dl, 2) an HbA1c between 39-46 mmol/mol (5.7–6.4%), or 3) a 2-hour oral glucose tolerance test between 140–199 mg/dl. T2D controls were defined as adults ≥40 years old who did not meet the definitions of T2D or prediabetes. Some studies, however, used their own in-house definitions and did not identify individuals with prediabetes. Definitions used in the additional biobanks and cohorts were similar to those used in PAGE are summarized in the Supplemental Information.<br><br>Variants were excluded if the imputation info score was <0.4 or if the effective sample size was less than 30.
Replication	The polygenic risk score (PRS) was first constructed and its performance evaluated in PAGE, then replicated across 13 additional biobanks and cohorts that were not included in the discovery GWAS summary statistics of the top-performing T2D PRS. Results from PAGE and these 13 biobanks and cohorts were meta-analyzed using inverse variance-weighted fixed-effect models within each population to obtain population-specific estimates.
Randomization	Randomization is not relevant for our study since all studies included are either longitudinal cohorts or biobanks.
Blinding	Blinding is not relevant for our study.

Reporting for specific materials, systems and methods

We require information from authors about some types of materials, experimental systems and methods used in many studies. Here, indicate whether each material, system or method listed is relevant to your study. If you are not sure if a list item applies to your research, read the appropriate section before selecting a response.

Materials & experimental systems

n/a	Involved in the study	
☒	☐	Antibodies
☒	☐	Eukaryotic cell lines
☒	☐	Palaeontology and archaeology
☒	☐	Animals and other organisms
☒	☐	Clinical data
☒	☐	Dual use research of concern
☒	☐	Plants

Methods

n/a	Involved in the study	
☒	☐	ChIP-seq
☒	☐	Flow cytometry
☒	☐	MRI-based neuroimaging

Plants

Seed stocks	*Report on the source of all seed stocks or other plant material used. If applicable, state the seed stock centre and catalogue number. If plant specimens were collected from the field, describe the collection location, date and sampling procedures.*
Novel plant genotypes	*Describe the methods by which all novel plant genotypes were produced. This includes those generated by transgenic approaches, gene editing, chemical/radiation-based mutagenesis and hybridization. For transgenic lines, describe the transformation method, the number of independent lines analyzed and the generation upon which experiments were performed. For gene-edited lines, describe the editor used, the endogenous sequence targeted for editing, the targeting guide RNA sequence (if applicable) and how the editor was applied.*
Authentication	*Describe any authentication procedures for each seed stock used or novel genotype generated. Describe any experiments used to assess the effect of a mutation and, where applicable, how potential secondary effects (e.g. second site T-DNA insertions, mosiacism, off-target gene editing) were examined.*
